# Failure identification of metastasis and non-sentinel lymph node metastasis in early gastric cancer

**DOI:** 10.3389/fonc.2026.1636493

**Published:** 2026-04-16

**Authors:** Huiliang Zhang, Qingwen Huang, Jitao Du, Xiangbin Wan

**Affiliations:** Department of General Surgery, The Affiliated Cancer Hospital of Zhengzhou University & Henan Cancer Hospital, Zhengzhou, China

**Keywords:** early gastric cancer, FFPE, frozen section, lymph node metastasis, sentinel lymph node biopsy

## Abstract

**Background:**

In the realm of sentinel lymph node navigation surgery for cancer patients, the intraoperative pathological examination of sentinel lymph nodes is of paramount importance in delineating the extent of surgical intervention. This study aimed to analyze the feasibility of frozen section of sentinel lymph node in early gastric cancer.

**Methods:**

A retrospective analysis of data prospectively collected was undertaken from a tertiary hospital. Patients with early gastric cancer were treated by sentinel lymph node biopsy followed by lymph node dissection. Primary outcome variables were the failure rate of frozen section identification and the incidence of metastasis in non-sentinel lymph nodes.

**Results:**

Of the 238 patients who underwent sentinel lymph node pelvic dissection, 30 were identified as having lymph node metastasis upon permanent pathological examination. Thirteen patients exhibited macrometastasis confirmed in both frozen and formalin-fixed and paraffin-embedded (FFPE) sections. Patients with negative sentinel lymph nodes in frozen sections but evidence of micrometastasis in FFPE sections did not experience lymph node recurrence during the follow-up period. In instances where tumor-positive lymph nodes were identified in frozen sections, metastasis in non-sentinel lymph nodes was detected in the paraffin blocks (8.3%, 2/24).

**Conclusions:**

The single-section hematoxylin and eosin staining method proves efficacious in detecting macrometastatic tumors through intraoperative pathological examination. Should the frozen section result be definitively negative, sentinel basin dissection can be executed with safety. Conversely, in the absence of such confirmation, the standard surgical approach is advisably pursued.

## Introduction

Due to advancements in healthcare initiatives, a significant number of early gastric cancers (EGCs) are now being detected and confirmed, particularly in countries with robust screening programs like Japan, where the early detection rate is notably higher (1). Radical excision is likely to be performed, but lymph node metastasis only develops with an incidence of about 15% (2). More reasonable options are required. Similarly to its application in managing oral cancer and skin cancer (3, 4), sentinel lymph node biopsy is also a viable option for EGC (3, 4). Sentinel lymph nodes are typically the first to receive lymphatic drainage from a tumor and are often the initial sites to exhibit metastasis. These nodes can be detected via visual inspection and the use of radioactive probes, and the absence of metastasis in these nodes obviates the need for extensive surgical intervention. Nonetheless, the sensitivity of sentinel lymph node biopsy is compromised in the context of gastric cancer due to the stomach’s multi-directional lymphatic flow (5). Consequently, sentinel lymph node pelvic resection has been employed, entailing the comprehensive excision of the lymphatic region encompassing both sentinel and adjacent lymph nodes to decrease the rate of false-negative results.

Recent findings from the SENORITA trial (6) suggest that combining laparoscopic sentinel pelvic resection with gastric conserving surgery leads to improved long-term survival rates and quality of life for patients with EGC, as compared to traditional laparoscopic standard gastrectomy (LSG) and lymph node dissection. If intraoperative frozen section analysis during sentinel lymph node navigation surgery (SNNS) confirms the presence of lymph node metastasis, it is crucial to proceed with standard gastrectomy and lymph node dissection. Hence, the intraoperative assessment of sentinel lymph node metastasis is essential in delineating the suitable extent of the surgical intervention. Despite the variability in accuracy, intraoperative frozen sections are widely recognized for their clinical value, with some studies indicating their accuracy can be as high as 95% or more. However, the debate continues as to whether they are the quintessential intraoperative diagnostic modality, given the potential for variability and the need for further research to standardize their use. Advanced techniques, including immunohistochemical (IHC) staining, reverse transcription polymerase chain reaction, and one-step nucleic acid amplification (7, 8). Nonetheless, these methodologies have not gained widespread traction due to their laborious nature, extensive temporal demands, and challenges associated with hyper-sensitivity, which are common issues in various fields that require strategic approaches to overcome.

Concealed metastasis refers to lymph node metastasis that evades detection by conventional diagnostic procedures, necessitating the use of additional investigative modalities. In the context of SNNS, the term ‘occult metastasis’ refers to lymph node metastasis that evades detection during intraoperative frozen section analysis but is later identified through comprehensive histological examination. The clinical import of occult metastasis has been a point of contention in diverse scholarly reports (9–11). Considering the findings from studies on micrometastases post-radical gastrectomy and lymph node dissection, as well as insights from research on occult metastases in breast cancer, the clinical implications of such metastases following SNNS warrant further investigation.

Thus, the aim of the current study is to ascertain the rate of identification failure in intraoperative frozen sections and the rate of non-sentinel lymph node metastasis in patients who underwent laparoscopic SNNS, to examine the rate of lymph node recurrence in patients who underwent laparoscopic sentinel lymph node assessment as an integral component of stomach-sparing surgical intervention.

## Patients and methods

### Ethical approval

This study received approval from Henan Cancer Hospital Institutional Research Committee, and written informed consent for medical research was obtained from all patients prior to the initiation of treatment. All methodologies were executed in accordance with the Declaration of Helsinki.

### Study design

To address our purpose, a retrospective analysis was conducted on patients who were prospectively enrolled. Between January 2015 and December 2022, a total of 238 consecutive patients were prospectively enrolled. Patients must met the following criteria: histologically confirmed gastric adenocarcinoma with clinical stage T1N0M0 (8th AJCC edition); tumor size <3 cm; tumor located ≥2 cm from both the pylorus and esophagogastric junction; and all histological subtypes were eligible. Exclusion criteria included multifocal disease, other malignancies within five years, previous gastric surgery, or neoadjuvant therapy. All these patients underwent SNNS followed by standard lymph node dissection no matter what the sentinel lymph node was positive or negative pathologically during frozen section. Information regarding demography, pathology, treatment, and follow-up was extracted.

### Study variable

**Figure 1:**
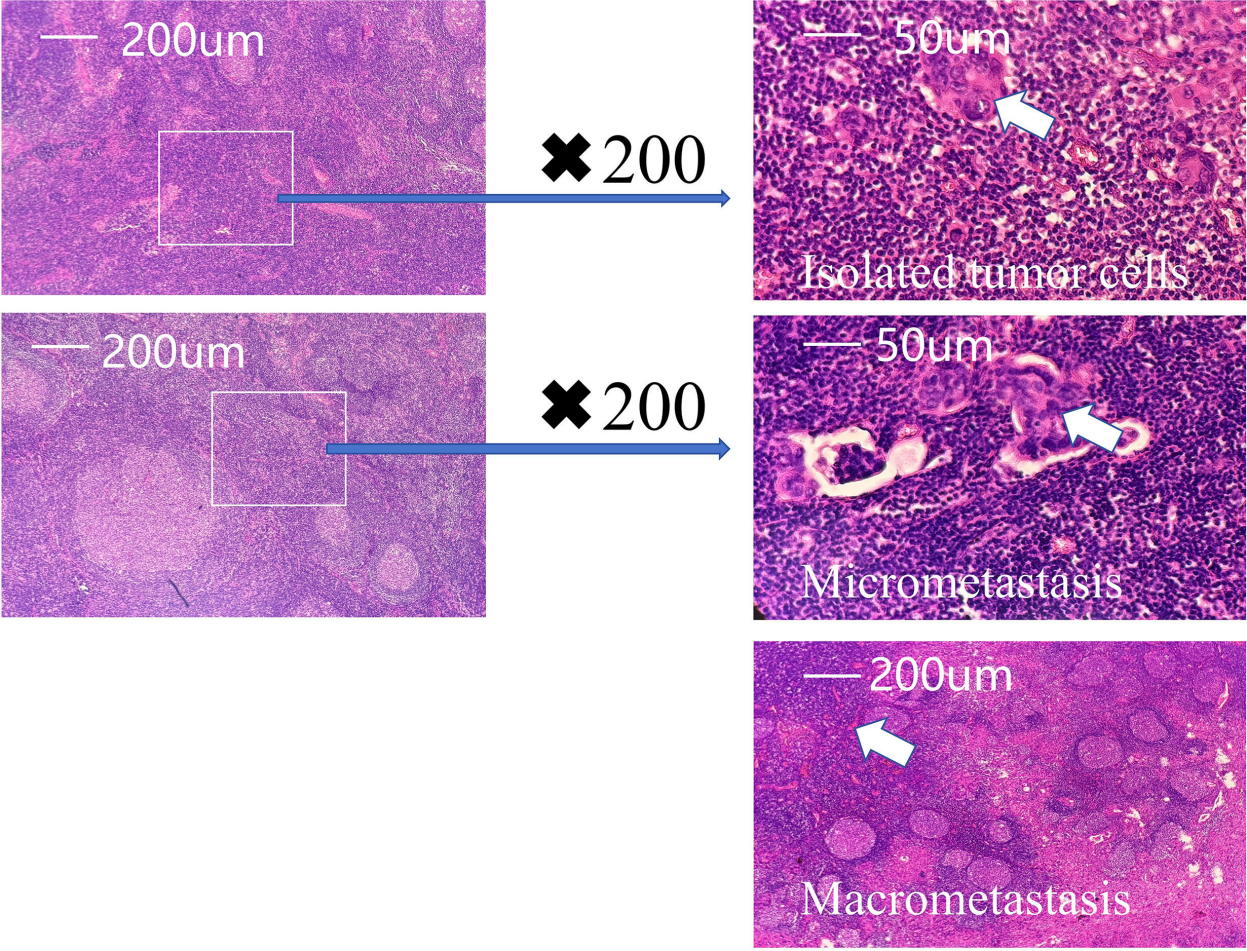
Intraoperative and postoperative pathological assessment of sentinel lymph nodes in early gastric cancer. (White arrow represents metastatic site).

The tumor and neck stages were classified according to the detailed criteria of the 8th edition of the AJCC staging system, which provides a comprehensive framework for the classification of solid cancers. In the context of cancer staging, the metastatic size in positive lymph nodes is categorized into isolated tumor cells, micrometastases, and macrometastases. Isolated tumor cells (ITCs) were defined as clusters of tumor cells ≤ 0.2 mm or single cells; micrometastases were defined as tumor deposits > 0.2 mm but ≤ 2.0 mm; and macrometastases were defined as tumor deposits > 2.0 mm (**Figure 1**).

### Sentinel lymph node

During the surgical procedure, a dual tracer consisting of indocyanine green and radiolabeled human serum albumin was endoscopically administered to the submucosal layer of the tumor, with 1 mL of each tracer injected into each of the four quadrants. Following a 15-minute latency post-injection, the gastric, splenic, and abdominal regions were visually scrutinized for the presence of dye, evidenced by green nodules, and assessed for radioactivity using a gamma probe, indicative of hot nodules. The sentinel basin, encompassing both hot and/or green lymph nodes along with the adjacent lymphatic systems, was meticulously excised from the surrounding tissues, ensuring a 1 cm margin around these lymph nodes. The excised basin was then retrieved and dissected into individual lymph nodes within the operating theater. These isolated lymph nodes were subsequently forwarded to pathologists for frozen section analysis to determine the presence of tumors, intraoperative immunohistochemistry was selectively performed (6, 11).

### Intraoperative and postoperative pathologic analysis

All sentinel pelvic nodes procured from the laparoscopic SNNS cohort were bisected with a solitary representative incision traversing the maximal plane for nodes ≤4 mm in diameter, a method distinct from the 2 mm interval serial sections along the maximum axis utilized in oncological cases. For nodes larger than 4 mm, transverse sections were created at 2 mm intervals parallel to the longitudinal axis. These frozen sections were assessed intraoperatively with hematoxylin and eosin (HE) staining. Should sentinel lymph nodes test positive or negative in the frozen sections, irrespective of the metastatic lesion’s dimensions, a standard gastrectomy and lymph node dissection were imperative, adhering to treatment protocols. Non-sentinel lymph nodes were those lymph nodes not included within the sentinel lymph node group, as identified following standard gastrectomy and lymph node dissection.

Post-surgical reevaluation of all sentinel lymph nodes was conducted to ascertain the presence of occult metastases. Formalin-fixed and paraffin-embedded (FFPE) tissue sections were subjected to a single HE staining and a cytokeratin immunohistochemistry. Moreover, they were sectioned at 200μm intervals for three deeper levels and examined using HE staining. The detection of macroscopic metastases in FFPE sections via enhanced methodologies necessitated patients to undergo standard gastrectomy and lymph node dissection. In contrast, if only microscopic metastases or isolated tumor cells were confirmed in the FFPE sections, the patients were closely monitored. Following the identification of metastasis in FFPE sections, a retrospective review of the HE slides from the intraoperative frozen examination was performed to corroborate the accuracy of the frozen diagnosis.

### Follow-up protocol

After discharge, all patients were followed up at our outpatient clinic according to a standardized protocol. Follow-up visits were scheduled every 3 months for the first 2 years, every 6 months for years 3 through 5, and annually thereafter. At each visit, a physical examination, laboratory tests (including tumor markers), and imaging studies (abdominopelvic computed tomography and chest radiography) were performed. Upper gastrointestinal endoscopy was conducted annually. Recurrence was confirmed by imaging findings and, when clinically indicated, by biopsy.

### Statistic analysis

Continuous variables are presented as means with standard deviations or as medians with interquartile ranges, depending on the normality of the data distribution. Categorical variables are expressed as frequencies and percentages. For group comparisons, the Chi-square test or Fisher’s exact test was used for categorical variables, as appropriate. Continuous variables were compared using the independent t-test or Mann–Whitney U test based on data distribution. All statistical tests were two-sided, and a p-value of less than 0.05 was considered statistically significant. Statistical analyses were performed using R software (version 3.4.4; R Foundation for Statistical Computing, Vienna, Austria).

## Results

### Baseline data

In total, 238 patients were analyzed with a mean age of 60 ± 10 years. There were 139 male and 99 female. The majority of patients underwent laparoscopic distal gastrectomy. Regarding tumor location, 18 were in the upper region, 131 in the middle, and 89 in the lower. The mean tumor size was 2.0 ± 0.5cm. In the 178 patients diagnosed with tubular adenocarcinoma, the differentiation levels were as follows: 28 cases were well differentiated, 57 cases were moderately differentiated, and 93 cases were poorly differentiated. Signet-ring cell carcinoma was diagnosed in 60 patients. Pathologic tumor stage was T1a in 99 patients, T1b in 106 patients, T2/3 in 33 patients. Lymph node metastasis occurred in 30 patients, in whom 23 cases were staged as N1, 4 cases staged as N2, and 3 cases staged as N3a. Patients with lymph node metastasis are more likely to be at a higher tumor stage (p=0.003). The median follow-up period for the entire cohort was 48 months (interquartile range: 36-72 months). (**Table 1**).

**Table 1:** Baseline data of the enrolled patients.

Variable	Entire	Lymph node metastasis (n=30)	No lymph node metastasis (n=208)	p
Age				
≤60	151	19	132	
>60	87	11	76	0.989
Sex				
Male	139	18	121	
Female	99	12	87	0.849
Resection extent*				
LWR	71	9	62	
LSR	8	1	7	
LDG	137	17	120	
LTG	16	2	14	
LPPG	6	1	5	1.000
Tumor location				
Upper	18	2	16	
Middle	131	18	113	
Lower	89	10	79	0.870
Tumor size (cm)				
≤2	106	16	90	
>2	132	14	118	0.330
Histology				
Tubular	178	21	157	
Well	28	4	24	
Moderate	57	8	49	
Poor	93	9	84	
Signet ring cell	60	9	51	0.873
Tumor stage				0.003
T1a	99	8	91	
T1b	106	12	94	
T2/3	33	10	23	
Lymph node metastasis (AJCC 8th N stage)				
N1 (1-2 regional LNs)	–	23	–	
N2 (3-6 regional LNs)	–	4	–	
N3a (7-15 regional LNs)	–	3	–	

*LWR, laparoscopic wedge resection; LSR, laparoscopic segmental resection; LDG, laparoscopic distal gastrectomy; LPPG, laparoscopic pylorus-preserving gastrectomy.

### Failure of identification in frozen sections

Among the 13 patients presenting with substantial metastases, two individuals were initially adjudged to have negative sentinel lymph nodes based on intraoperative frozen section reports. Nevertheless, a subsequent re-examination of the frozen HE slides uncovered significant metastatic involvement, thereby corroborating an initial misdiagnosis. Consequently, all instances of significant metastasis were discernible in the frozen sections, yielding a recognition failure rate of 0%.

Of the 17 patients exhibiting micrometastasis within formalin-fixed, paraffin-embedded specimens, 10 were erroneously classified as negative through intraoperative frozen section analysis. Discounting the confirmed micrometastasis in sentinel lymph nodes identified during the review of frozen slides from four patients, micrometastasis remained undetected in the frozen sections of six patients, culminating in a detection failure rate of 35.3%.

### Diagnostic accuracy of frozen section

Among the 238 patients, frozen section correctly identified 24 of the 30 patients with confirmed lymph node metastasis, yielding a sensitivity of 80.0% (95% confidence interval [CI]: 61.4-92.3%). All 208 patients without lymph node metastasis were correctly identified on frozen section, resulting in a specificity of 100% (95% CI: 98.2-100%). The false-negative rate was 20.0% (95% CI: 7.7-38.6%), and the negative predictive value was 97.2% (95% CI: 94.1-98.7%). The positive predictive value was 100% (95% CI: 85.8-100%) as there were no false-positive findings. Importantly, all six false-negative cases occurred in patients with micrometastasis (mean tumor size 0.7 mm; range 0.5-1.0 mm) that were not detected on the single-section HE staining during intraoperative assessment. When analysis was restricted to macrometastases (>2.0 mm), frozen section demonstrated 100% sensitivity (13/13) with no false-negative results. (**Table 2**).

**Table 2:** Diagnostic accuracy of intraoperative frozen section for sentinel lymph node metastasis.

Overall diagnostic performance (N=238 patients)			
Parameter	Formula	Value	95% Confidence interval
True Positive (TP)	Frozen + / Final +	24	—
True Negative (TN)	Frozen - / Final -	208	—
False Positive (FP)	Frozen + / Final -	0	—
False Negative (FN)	Frozen - / Final +	6	—
Sensitivity	TP / (TP + FN)	80.0%	61.4-92.3%
Specificity	TN / (TN + FP)	100%	98.2-100%
False-Negative Rate	FN / (TP + FN)	20.0%	7.7-38.6%
Negative Predictive Value	TN / (TN + FN)	97.2%	94.1-98.7%
Positive Predictive Value	TP / (TP + FP)	100%	85.8-100%
Accuracy	(TP + TN) / Total	97.5%	94.6-99.1%
Stratified Analysis by Metastasis Type			
Metastasis Category	Detection Rate	Sensitivity	False-Negative Rate
Macrometastasis (>2.0 mm)	13/13	100%	0%
Micrometastasis (≤2.0 mm)	11/17	64.7%	35.3%
Small micrometastasis (<1.5 mm)	8/14	57.1%	42.9%
Large micrometastasis (1.5-2.0 mm)	3/3	100%	0%

### Lymph node recurrence

Of the 238 patients, lymph node recurrence was documented in three individuals. Following the successful salvage of recurrent disease, one patient experienced a favorable outcome with adjuvant chemotherapy, and the other two patients survived for over three years following the surgical intervention.

### Lymph node metastasis in sentinel and non-sentinel nodes

Patients were systematically classified based on the presence and extent of lymph node metastasis in the sentinel pelvic lymph nodes. Six patients exhibited micrometastases undetectable in frozen sections but identified in FFPE sections. The mean diameter of the sentinel lymph node tumors in this cohort was 0.7 millimeters. Notably, patients in this category did not encounter any lymph node recurrences during the entire surveillance period (0%, 0/6). The second cohort comprised 11 cases where tumors were identified in both frozen sections and exhibited micro metastases in FFPE tissue. This group’s average tumor size was 1.1 millimeters, which is considered small and typically associated with a lower metastasis rate. However, the non-sentinel lymph node metastasis rate observed was 9.1% (1/11), indicating that even small tumors can exhibit metastatic behavior. The histogram depicting metastasis size for the second group revealed two disparate populations. Therefore, we further divided this group into two subgroups: small metastasis and large metastasis, with the latter subgroup showing involvement in non-sentinel lymphatic drainage areas. The third group was characterized by tumors that tested positive in the frozen examination and displayed macrometastases within the paraffin blocks, with an average tumor size of 3.6 millimeters; the non-sentinel lymph node metastasis rate within this group stood at 7.7% (1/13). (**Table 3**, **Supplementary Table 1**).

**Table 3:** Lymph node (LN) metastasis in sentinel and non-sentinel lymph node.

Category	Number of patients/retrieved sentinel LN	LN metastasis in non-sentinel LN
FF-negative and FFPE-micrometastasis*	6/11	0%
FF-positive and FFPE-micrometastasis	11/10	9.1% (1/11)
Small metastasis	8/9	0%
Large metastasis	3/11	33.3% (1/3)
FF-positive and FFPE-macrometastasis	13/21	7.7% (1/13)

*FF, fresh frozen; FFPE, formalin-fxed parafn-embedded.

## Discussion

The present study provides important insights into the clinical utility of intraoperative frozen section analysis during sentinel lymph node navigation surgery for early gastric cancer. Our most important finding was that intraoperative frozen examination in identifying macrometastases was impeccable, with a failure rate of zero. Six patients, who were deemed tumor-free following cryo-examination but later exhibited micrometastasis in FFPE samples, did not suffer lymph node recurrence during follow-up. Sentinel lymph node analysis via frozen sections revealed positivity, while FFPE sections uncovered micro- or macrometastases in the patients. The rates of lymph node metastasis in non-sentinel pelvic lymph nodes were 9.1% and 7.7% for micro- and macrometastases, respectively, suggesting that even small tumor deposits in sentinel nodes carry a non-negligible risk of further nodal involvement.

Intraoperative diagnosis in SNNS is of paramount importance for the detection of sentinel lymph node metastasis. The majority of prior studies have evaluated sentinel lymph nodes intraoperatively using frozen sections stained with hematoxylin and eosin. The false-negative rate for HE-stained frozen sections has been reported to be as high as 50% in some series (12). Techniques such as rapid immunohistochemistry, serial sectioning, and one-step nucleic acid amplification have been introduced to augment sensitivity and diminish false-negative rates compared to the conventional frozen HE method (7, 8). Nevertheless, these approaches have not been widely adopted in routine clinical practice due to their labor-intensive and time-consuming characteristics, as well as the risk of precipitating unnecessary gastrectomies because of heightened sensitivity. The challenge lies in balancing diagnostic accuracy against practical feasibility in the operating room setting.

In this study, lymph nodes were sliced along their largest plane and then stained using the traditional and straightforward intraoperative HE staining technique. The identification of all macrometastases was achieved through the examination of representative sections of frozen tissues, which were subsequently subjected to HE staining, a widely recognized technique in pathology for the visualization of cellular and structural details. Unfortunately, during the interpretation process, two cases of macrometastasis in the frozen sections were initially missed, emphasizing the importance of thorough evaluation and the potential value of second reads or quality assurance protocols in intraoperative pathology. This observation underscores that even with macrometastases, diagnostic errors can occur due to sampling or interpretive factors, highlighting the need for continued education and standardization.

Conversely, a solitary HE-stained section proved inadequate for diagnosing all micrometastases intraoperatively, given the elevated rate of recognition failure (35.3%, 6/17). This limitation is well-recognized and has spurred the development of more sensitive, albeit less practical, techniques (7, 8). The clinical relevance of these occult micrometastases remains a subject of considerable debate (13). While some studies in gastric and other cancers suggest that micrometastases may have a prognostic impact, others, such as the IBCSG 23-01 trial in breast cancer, have demonstrated that they do not warrant additional surgical intervention like completion lymph node dissection (14). In the present study, the six patients with occult micrometastasis (mean size 0.7 mm) did not experience lymph node recurrence during a median follow-up of 48 months. This finding suggests that, within this timeframe and for this specific size range, these micrometastases were likely confined to the sentinel basin, with no clinically significant involvement of the unexcised, non-sentinel lymph nodes. This aligns with the hypothesis that very small tumor deposits may be biologically indolent or adequately controlled by local immune responses, as suggested by recent work on the immunological microenvironment of sentinel nodes. However, while a median of 48 months is substantial, late recurrences from micrometastatic disease are a theoretical possibility. The natural history of untreated micrometastases in gastric cancer is not fully defined. Therefore, while our data are reassuring and support a strategy of vigilant monitoring for these specific patients, they do not definitively prove the long-term safety of this approach. The absence of recurrence to date must be interpreted with caution, and these patients continue to be followed according to a rigorous protocol. The optimal management of occult micrometastases will ultimately require confirmation from studies with longer-term follow-up and perhaps a pooled analysis of data from multiple prospective cohorts.

In the present study, patients exhibiting micrometastasis in FFPE specimens were categorized into small and large groups based on the dimensions of the lymph node metastases. The small-scale group mirrored Group 1 in size, with both being negative on frozen section but positive for micrometastasis on permanent pathology, and no additional metastases were found in the non-sentinel lymph nodes. Conversely, the large group manifested metastases in the non-sentinel lymph nodes. Analogous to macroscopic metastases, a larger number of microscopic metastases may increase the risk of non-sentinel lymph node metastasis. Yamamoto et al. (15) demonstrated that lymph node tumors with a diameter smaller than 1500 µm are associated with higher survival rates compared to those exceeding 1500 µm, aligning with our findings and the broader understanding that smaller tumor size often correlates with better prognosis. Consequently, further inquiry is imperative to ascertain a critical threshold below 2000 µm, which is presently employed as a criterion for delineating macroscopic metastases. Our data suggest that a threshold of approximately 1.5 mm may better discriminate between patients at risk for non-sentinel node involvement and those who can safely avoid additional dissection.

The immunological microenvironment of sentinel lymph nodes has emerged as a critical factor in understanding metastatic behavior and patient outcomes. Recent work by Sonoda et al. (16) provides valuable insights into the cellular composition of sentinel versus non-sentinel nodes in gastric cancer patients. Their immunohistochemical and morphometric analysis revealed that sentinel nodes without metastasis exhibited significantly smaller subcapsular dendritic cell clusters compared to non-sentinel nodes (p=0.037), suggesting that dendritic cells may migrate from the subcapsular sinus to paracortical areas upon antigen exposure. More importantly, they demonstrated a significantly larger overlap between DC-SIGN-positive dendritic cells and CD169-positive macrophages in sentinel nodes than in non-sentinel nodes (p=0.019), indicating enhanced cross-presentation of cancer antigens at the first site of lymphatic drainage. This “preconditioning” of the sentinel node may represent an early immunological response to tumor-derived antigens even before metastatic cells become histologically detectable. Conversely, in patients with established nodal metastasis, they observed markedly reduced areas of dendritic cells and CD169-positive macrophages within metastatic nodes, suggesting cancer-induced immune suppression even at early stages. These findings provide a biological rationale for the clinical observations in our study: the presence of micrometastases beyond a certain size threshold may reflect not only tumor burden but also failure of the local immune microenvironment to contain cancer cells, thereby increasing the risk of non-sentinel node involvement.

According to the SENORITA protocol (17), the detection of a tumor in the sentinel pelvic lymph nodes during intraoperative assessment necessitates the conversion of the surgical procedure to standard gastrectomy accompanied by radical lymph node dissection. This study indicates that in instances where tumor metastasis is identified in the frozen sections of sentinel basins, the potential for additional lymph node metastasis in non-sentinel basins cannot be dismissed. When the extent of lymph node metastasis remains indeterminate in frozen sections, standard gastrectomy coupled with lymph node dissection should be executed, irrespective of the size of the lymph node metastasis observed in the frozen sections. This conservative approach ensures oncological safety while acknowledging the limitations of intraoperative assessment.

Recent developments in function-preserving surgery have further expanded the potential applications of sentinel node navigation. The sealed endoscopic full-thickness resection technique described by Itoh et al. (18) represents an innovative approach combining SNNS with minimally invasive local resection. In their series of 16 patients with cT1 gastric cancer ineligible for endoscopic submucosal dissection, 12 (75%) were sentinel node-negative and proceeded to sealed EFTR, with no cases of metastasis or recurrence during a mean follow-up of 6.5 years. This technique addresses a critical concern in minimally invasive surgery: preventing peritoneal dissemination during full-thickness resection by covering the serosa with a silicone sheet before incision. The convergence of sentinel node navigation with advanced endoscopic techniques exemplifies the trend toward individualized, function-preserving surgery for early gastric cancer. However, as the authors note, limitations regarding tumor location (difficult application in the cardia, pylorus, or lesser curvature of the gastric angle) and the need for further case accumulation underscore that these approaches remain under investigation.

Following laparoscopic subtotal gastrectomy and lymph node dissection, three patients in our series experienced lymph node recurrence. In one instance, the tumor was situated on the greater curvature/anterior wall side, with the sentinel basin identified at lymph node station #4d. With the confirmation of no tumor cells in the frozen sections, the patient was diagnosed with lymph node recurrence (stations #6, #7, and #11p) one year postoperatively. This case may be considered as a failure in the detection of the sentinel basin during SNNS, possibly due to aberrant lymphatic drainage or incomplete basin dissection. Another patient underwent laparoscopic wedge resection and sentinel pelvic dissection, as frozen section analysis revealed no tumor cells in the sentinel pelvic region. Postoperatively, only isolated tumor cells were detected in the three deeper sections. The patient was diagnosed with retroperitoneal lymph node recurrence two years postoperatively and received palliative chemotherapy, surviving beyond three years. In this scenario, the possibility of skipped metastasis or misdiagnosed recurrence exists. However, considering that distant metastasis occurred in two patients within the standard surgery group, the lymph node recurrence rates were consistent with prior studies (17). Several key differences exist between our study and the SENORITA trial. First, our study focused specifically on the failure rate of frozen section identification and non-sentinel lymph node metastasis, while the SENORITA trial primarily evaluated long-term survival outcomes and quality of life. Second, our cohort (n=238) represents a single-institution experience with standardized pathological protocols, whereas the SENORITA trial was a multicenter randomized phase III trial. Third, our identification of a critical size threshold for micrometastases (approximately 1.5 mm) that may predict non-sentinel lymph node involvement extends the findings from the SENORITA trial, which did not specifically analyze this parameter. These complementary findings suggest that while the sentinel basin concept is valid, careful intraoperative assessment remains crucial for determining the extent of surgical resection. The integration of immunological markers, such as those described by Sonoda et al. (16), may in the future enhance our ability to predict which patients with sentinel node involvement require complete lymphadenectomy.

The diagnostic accuracy parameters reported in this study provide a comprehensive evaluation of intraoperative frozen section reliability for sentinel lymph node assessment in early gastric cancer. These findings align with previous reports, which reported false-negative rates ranging from 14% to 50% for HE-stained frozen sections (12). The perfect specificity (100%) in our series confirms that positive frozen section findings can reliably guide conversion to standard gastrectomy without risk of unnecessary extensive resection (19). The modest sensitivity reflects the inherent limitation of single-section HE staining for detecting micrometastases, as all false-negative cases in our series were micrometastases <1.0 mm. However, the high negative predictive value (97.2%) indicates that a negative frozen section result provides strong reassurance against missed clinically significant nodal involvement. This supports that sentinel basin dissection can be safely performed when frozen section is definitively negative. The 100% sensitivity for macrometastases (>2.0 mm) demonstrates that the technique is reliable for detecting tumor deposits that would mandate standard lymphadenectomy according to current treatment guidelines.

We acknowledge the limitations of this study. While the data were collected prospectively, the retrospective analysis is inherently subject to potential selection and analytical biases. Furthermore, as a single-center study conducted at a tertiary referral hospital, the findings may reflect specific institutional practices and patient populations, which could limit their generalizability to broader, multi-institutional settings. Future multi-center, prospective studies are warranted to validate our findings, particularly the proposed critical size threshold for micrometastases.

In summation, the extant research indicates that our pathological protocol is appropriately tailored for the assessment of sentinel lymph nodes. The use of single-slice HE staining is considered adequate for diagnosing macroscopic metastases during intraoperative histological evaluations; if latent micrometastases are identified during a more thorough postoperative evaluation, a program of vigilant monitoring would suffice. In light of the potential for metastasis to non-sentinel lymph nodes, sentinel lymph node dissection may be performed unless histological evidence of metastasis is found in the sentinel lymph nodes during the intraoperative assessment. Nonetheless, the identification of tumor metastasis in the sentinel basin through frozen sections mandates the execution of lymph node dissection and gastrectomy to ensure the thoroughness of cancer treatment, but longer-term surveillance is essential to definitively determine their clinical significance and to confirm the safety of omitting completion lymphadenectomy. Additional investigative efforts are required for the clinical application of SNNS to ascertain the critical diameter threshold for tumor detection in metastatic lymph nodes and to elucidate the adverse prognostic impact of isolated tumor cells. Future research should also focus on integrating immunological markers of sentinel node preconditioning and suppression, as these may provide complementary information to guide treatment de-escalation strategies. As techniques for minimally invasive, function-preserving resection continue to evolve in parallel with sentinel node navigation, the goal of truly individualized surgery for early gastric cancer moves closer to clinical reality.

## Data Availability

The original contributions presented in the study are included in the article/**Supplementary Material**. Further inquiries can be directed to the corresponding author.
