# Supplementary material for: Failure identification of metastasis and non-sentinel lymph node metastasis in early gastric cancer
Source: Front Oncol. 2026 Apr 16;16:1636493. doi: 10.3389/fonc.2026.1636493 (PMC13128356; doi:10.3389/fonc.2026.1636493)
Supplement: Supplementary file 1 [file Table1.doc]

### **Supplementary Table 1. Detailed Pathological Findings for 30 Patients with Lymph Node Metastasis**

| **Patient No.** | **Frozen Section**  **(FS) Diagnosis** | **Final FFPE Diagnosis** | **Metastasis Size Category** | ****Exact Metastasis Size****  ****(mm)**** | **Non-Sentinel LN Metastasis** | **IHC Status** | **Findings in 3 Deeper Sections**  **(from FFPE)** |
| --- | --- | --- | --- | --- | --- | --- | --- |
| **Group A: FS Negative → FFPE Micrometastasis (n=6, Mean Size = 0.7 mm)** |  |  |  |  |  |  |  |
| 1 | Negative | Micrometastasis | Micrometastasis | 0.5 | Negative | Positive | Confirmed Micrometastasis |
| 2 | Negative | Micrometastasis | Micrometastasis | 0.6 | Negative | Positive | Confirmed Micrometastasis |
| 3 | Negative | Micrometastasis | Micrometastasis | 0.6 | Negative | Positive | Confirmed Micrometastasis |
| 4 | Negative | Micrometastasis | Micrometastasis | 0.7 | Negative | Positive | Confirmed Micrometastasis |
| 5 | Negative | Micrometastasis | Micrometastasis | 0.8 | Negative | Positive | Confirmed Micrometastasis |
| 6 | Negative | Micrometastasis | Micrometastasis | 1.0 | Negative | Positive | Confirmed Micrometastasis |
| **Group B: FS Positive → FFPE Micrometastasis (n=11, Mean Size = 1.1 mm)** |  |  |  |  |  |  |  |
| **Subgroup B1: Small Metastasis (n=8, No non-sentinel LN involvement)** |  |  |  |  |  |  |  |
| 7 | Positive | Micrometastasis | Small Metastasis | 0.8 | Negative | Positive | Confirmed Micrometastasis |
| 8 | Positive | Micrometastasis | Small Metastasis | 0.9 | Negative | Positive | Confirmed Micrometastasis |
| 9 | Positive | Micrometastasis | Small Metastasis | 0.9 | Negative | Positive | Confirmed Micrometastasis |
| 10 | Positive | Micrometastasis | Small Metastasis | 1.0 | Negative | Positive | Confirmed Micrometastasis |
| 11 | Positive | Micrometastasis | Small Metastasis | 1.0 | Negative | Positive | Confirmed Micrometastasis |
| 12 | Positive | Micrometastasis | Small Metastasis | 1.1 | Negative | Positive | Confirmed Micrometastasis |
| 13 | Positive | Micrometastasis | Small Metastasis | 1.2 | Negative | Positive | Confirmed Micrometastasis |
| 14 | Positive | Micrometastasis | Small Metastasis | 1.2 | Negative | Positive | Confirmed Micrometastasis |
| **Subgroup B2: Large Metastasis (n=3, One with non-sentinel LN involvement)** |  |  |  |  |  |  |  |
| 15 | Positive | Micrometastasis | Large Metastasis | 1.6 | **Positive** | Positive | Confirmed Micrometastasis |
| 16 | Positive | Micrometastasis | Large Metastasis | 1.7 | Negative | Positive | Confirmed Micrometastasis |
| 17 | Positive | Micrometastasis | Large Metastasis | 1.8 | Negative | Positive | Confirmed Micrometastasis |
| **Group C: FS Positive → FFPE Macrometastasis (n=13, Mean Size = 3.6 mm)** |  |  |  |  |  |  |  |
| 18 | Positive | Macrometastasis | Macrometastasis | 2.5 | Negative | Positive | Confirmed Macrometastasis |
| 19 | Positive | Macrometastasis | Macrometastasis | 2.8 | Negative | Positive | Confirmed Macrometastasis |
| 20 | Positive | Macrometastasis | Macrometastasis | 3.0 | Negative | Positive | Confirmed Macrometastasis |
| 21 | Positive | Macrometastasis | Macrometastasis | 3.2 | Negative | Positive | Confirmed Macrometastasis |
| 22 | Positive | Macrometastasis | Macrometastasis | 3.4 | Negative | Positive | Confirmed Macrometastasis |
| 23 | Positive | Macrometastasis | Macrometastasis | 3.5 | Negative | Positive | Confirmed Macrometastasis |
| 24 | Positive | Macrometastasis | Macrometastasis | 3.6 | Negative | Positive | Confirmed Macrometastasis |
| 25 | Positive | Macrometastasis | Macrometastasis | 3.7 | Negative | Positive | Confirmed Macrometastasis |
| 26 | Positive | Macrometastasis | Macrometastasis | 3.9 | Negative | Positive | Confirmed Macrometastasis |
| 27 | Positive | Macrometastasis | Macrometastasis | 4.1 | Negative | Positive | Confirmed Macrometastasis |
| 28 | Positive | Macrometastasis | Macrometastasis | 4.2 | Negative | Positive | Confirmed Macrometastasis |
| 29 | Positive | Macrometastasis | Macrometastasis | 4.5 | Negative | Positive | Confirmed Macrometastasis |
| 30 | Positive | Macrometastasis | Macrometastasis | 5.0 | **Positive** | Positive | Confirmed Macrometastasis |
